# Spatially Distributed Dendritic Resonance Selectively Filters Synaptic Input

**DOI:** 10.1371/journal.pcbi.1003775

**Published:** 2014-08-21

**Authors:** Jonathan Laudanski, Benjamin Torben-Nielsen, Idan Segev, Shihab Shamma

**Affiliations:** 1 Scientific and Clinical Research Department, Neurelec, Vallauris, France; 2 Equipe Audition, Département d'études cognitives, Ecole Normale Supérieure, Paris, France; 3 Computational Neuroscience Unit, Okinawa Institute of Science and Technology, Okinawa, Japan; 4 Department of Neurobiology and the Edmond and Lily Safra Center for Brain Science, Hebrew University of Jerusalem, Jerusalem, Israel; 5 Institute for Systems Research and Department of Electrical & Computer Engineering, University of Maryland, College Park, Maryland, United States of America; Universitat Pompeu Fabra, Spain

## Abstract

An important task performed by a neuron is the selection of relevant inputs from among thousands of synapses impinging on the dendritic tree. Synaptic plasticity enables this by strenghtening a subset of synapses that are, presumably, functionally relevant to the neuron. A different selection mechanism exploits the resonance of the dendritic membranes to preferentially filter synaptic inputs based on their temporal rates. A widely held view is that a neuron has one resonant frequency and thus can pass through one rate. Here we demonstrate through mathematical analyses and numerical simulations that dendritic resonance is inevitably a spatially distributed property; and therefore the resonance frequency varies along the dendrites, and thus endows neurons with a powerful spatiotemporal selection mechanism that is sensitive both to the dendritic location and the temporal structure of the incoming synaptic inputs.

## Author Summary

Neurons are constantly bombarded by thousands of inputs. Synaptic plasticity is generally accepted as a mechanism to select certain inputs by strengthening their synapses while reducing the effects of others by weakening them. Another biophysical mechanism to select inputs is through membrane resonance that enhances neuronal response to inputs arriving at a specific temporal rate while reducing others. In the classical view, a neuron has one such resonance frequency at which inputs can be preferentially filtered. By dissecting the biophysical mechanism underlying neuronal resonance we find that neurons in fact express a wide range of resonance frequencies spatially distributed along their dendrites. We further show that such dendritic resonance can endow a neuron with a true spatio-temporal filtering property of its inputs: neurons can preferentially filter inputs based on their dendritic location and/or temporal signature. We speculate that this new insight has pivotal consequences for learning and plasticity.

## Introduction

Neurons are constantly bombarded by thousands of synaptic inputs, so it is essential that neurons are able to listen *selectively* to subsets of these inputs. Throughout the sensory pathways, topographic maps ensure that neurons are able to sample a limited range of the stimulus space [1]. But the use of space is only one means by which input selectivity is achieved in the central nervous system. Another effective means is to respond selectively to particular temporal input patterns. A range of mechanisms can facilitate temporal selectivity ranging from pre-synaptic short-term plasticity [2]–[6], learning strategies of specific temporal patterns [7]–[10], to post-synaptic membrane resonances which enhance responses to specific input rates [11]–[13].

The focus of this study is the latter mechanism of resonance, membrane resonance, which has been traditionally considered a scalar property of a neuron: one neuron has one preferred resonance frequency [11], [14]. This view, however, is inconsistent with the increasing awareness of the complexity of dendritic ramifications, the non-uniform spatial distribution of their ionic channels and highly localized non-linearities. Such elaborate biophysics can endow single neurons with multiple resonances occuring at a wide range of frequencies and bandwidths, and thus enable neurons to act as multi-dimensional input classifiers. Here, we explore this idea using both analytic methods and numerical simulations of neurons with both simplified and realistic dendritic structures. We show how spatial profiles of resonance frequencies emerge naturally in dendrites, facilitating selective filtering of synaptic inputs based on their location and temporal signature. Our findings thus counter the widely-held assumption that input selection is based on a single prefered frequency band regardless the location of the synaptic input.

## Results

### Origin of 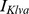 resonance in membrane and dendrites

**Figure 1:**
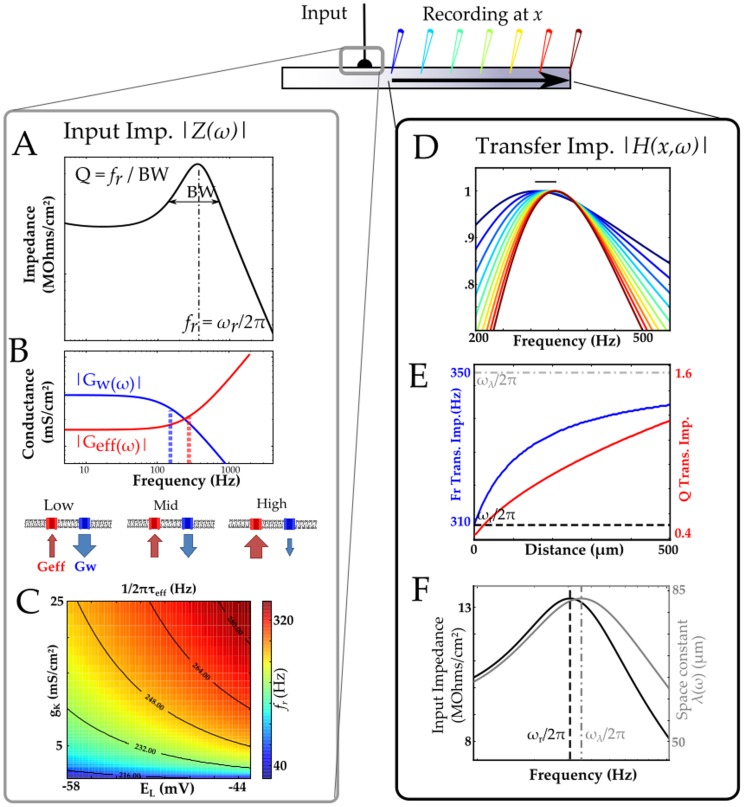
Resonance frequency in a cylindrical cable model. A. Input impedance and definition of resonance frequency (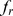) and resonance sharpness (Q-factor). B. Biophysical properties underlying resonance. A resonance is obtained if the effective admitttance 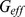 increases at higher frequencies (dotted red line) than the decrease of 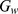 (dotted blue line). C. Range of resonance frequencies, 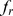, of the input impedance ensuing from a realistic range of leak reversal potenial (E_L_) and potassium conductance density 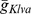. 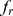 is color coded while isobars indicates the effective cut-off frequency (red dotted line in B). The resonance is set by the effective cut-off frequency 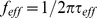 (black contour line) which depends on the potassium conductance density (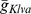) and effective reversal potential of the membrane (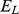); 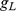 is kept constant at 1 mS/cm2. D. Normalized transfer impedance of a semi-infinte cable measured at different position along the cable with positions color-coded (as in the schematics above). The range of resonance frequencies (310–340 Hz) expressed by the cable is displayed as an horizontal bar. E. The resonance of the membrane patch is different from the resonance frequency of the space constant. This inhrent mismatch produces the gradual change toward higher frequencies as distance between the recording and input sites increases F. The spatial profile of resonance frequency (blue solid line – left ordinate axis) best displays how 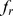 varies along the cable and is bounded by the resonance frequency of the input impedance (lower horizontal blue dotted line) and the resonance frequency of the space constant (upper horizontal blue dash-dotted line). The spatial profile of Q-factor is displayed as a red solid line (right ordinate axis). Both the membrane patch (A,B and C) and cable models (D and E) consist of a leak current, fast potassium current 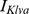 and static H-type current 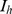 (see Methods).

Resonance in neuronal membranes has been described by many experimentalists and theoreticians [11], [12], [15]–[18]; it requires an interplay between at least two conductances with different dynamics. Figure 1A illustrates how an interaction between a membrane's passive electrical properties (resistance and capacitance) and one voltage-dependent current (low voltage-activated potassium current, 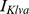) can give rise to a resonant membrane impedance (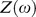) comprised of two admittances: 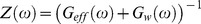. The interplay between these admittances produces the impedance resonance in much the same way as the restorative and regenerative conductances interact to form a resonance. The first admittance, 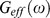, is an effective leak (red curve in Figure 1B) that is mostly associated with the classic membrane passive RC-circuit (time-constant 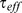; see *METHODS*), and which acts as a shunt at high frequencies as schematically illustrated by the large red arrow below the plots. The second admittance 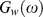 (blue curve in Figure 1B) is due to the 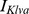 channels whose limited activation rate (time-constant 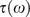) leaves them increasingly closed at frequencies higher than 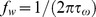, as depicted by the small blue arrow at right. The sum of these two admittances often results in a minimum at a mid frequency range producing a peak impedance 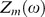 at a resonance frequency 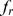 (Figure 1A). This minimum occurs when the increase in 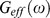 counter-balances the drop of 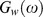. Since the increase of 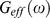 takes place for frequencies higher than 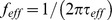, the resonance frequency 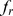 is always higher than 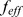. This is demonstrated in figure 1C where 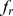 is color-coded for different values of 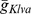 and 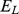 while 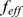 is displayed as black line contours. Clearly, the resonance frequency 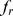 and its sharpness (*Q*) depend on 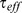, 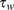, 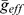 and 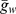, and through them on any biophysical parameters affecting the resting state of the membrane. As such, 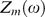 is affected by the reversal potential 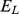, membrane leak conductance 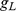, and maximal potassium conductance 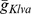 (see *METHODS*, *Figure 1C* and *Supplementary Figure S1*). As shown in Figure 1C, the resonance frequency increases monotonically both with increasing potassium channel density 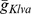 and with its steady state level (set by 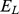). The sharpness of tuning Q depends on how much 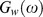 can decrease before the increase in 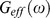 takes place and on how close in frequency these two changes occur. Hence, the dependence of Q upon the biophysical parameters is complex. For instance, Supplementary figure S1 A2 illustrates how changes in the leak conductance 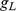 produces nonmonotonic changes in Q. To conclude, even in an isopotential patch of membrane with a linearized model of channel dynamics, the resonance frequency 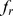 can vary substantially (300% or 120<
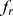
<350 Hz) depending on a range of parameter values typically found at different locations of a dendrite (Figure 1C and Supplementary Figure S1).

A key objective of our study is to explore the influence of “space” (namely dendritic location) on the resonance properties. To do so, we distinguish between local *input impedance*, 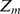, and the *transfer impedance* 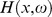, that is the total transfer function between the input at location x and a recording electrode at the soma, as illustrated in Figure 1D. It has been shown [19] that if the membrane impedance 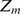 is bandpass, then so are the transfer impedance and the cable space constant 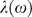, a measure of the electrical compactness of the dendrite. Computing the transfer impedance using just a uniform membrane model already reveals a strong spatial profile of resonance frequencies as illustrated in Figure 1D (see *METHODS*). This dependence arises mostly from an inherent mismatch 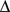 between the resonance of the input impedance (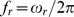) and that of the space constant (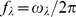) as shown in figure 1E. By definition the space constant 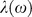 and the input impedance 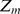 are related (see Methods) and the mismatch 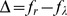, which is influenced by 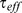, 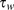, 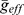 and 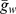, is non-zero for a large set of parameters (i.e. 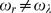; see Supplementary Figure S1 B1). This implies that in most cases, a spatial profile of resonance frequencies emerges along the semi-infinite cable: When the injection and recording site are close to one another, the resonance frequency of the transfer impedance is mostly that of the input impedance 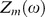. With increasing distance between both sites, the resonance frequency of the transfer impedance becomes more influenced by the resonance frequency of the frequency-dependent space constant 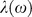. Figure 1F illustrates this effect and demonstrates that with plausible parameters the resonance frequency of the transfer impedance can change by as much as 11% over just the first 500 µm (of a semi-infinite cable model). Thus, the mere spatial extent of a dendrite already results in a spatially distributed profile of resonant frequencies.

### Dendritic morphology, non-uniform ionic channel distribution and boundary conditions

**Figure 2:**
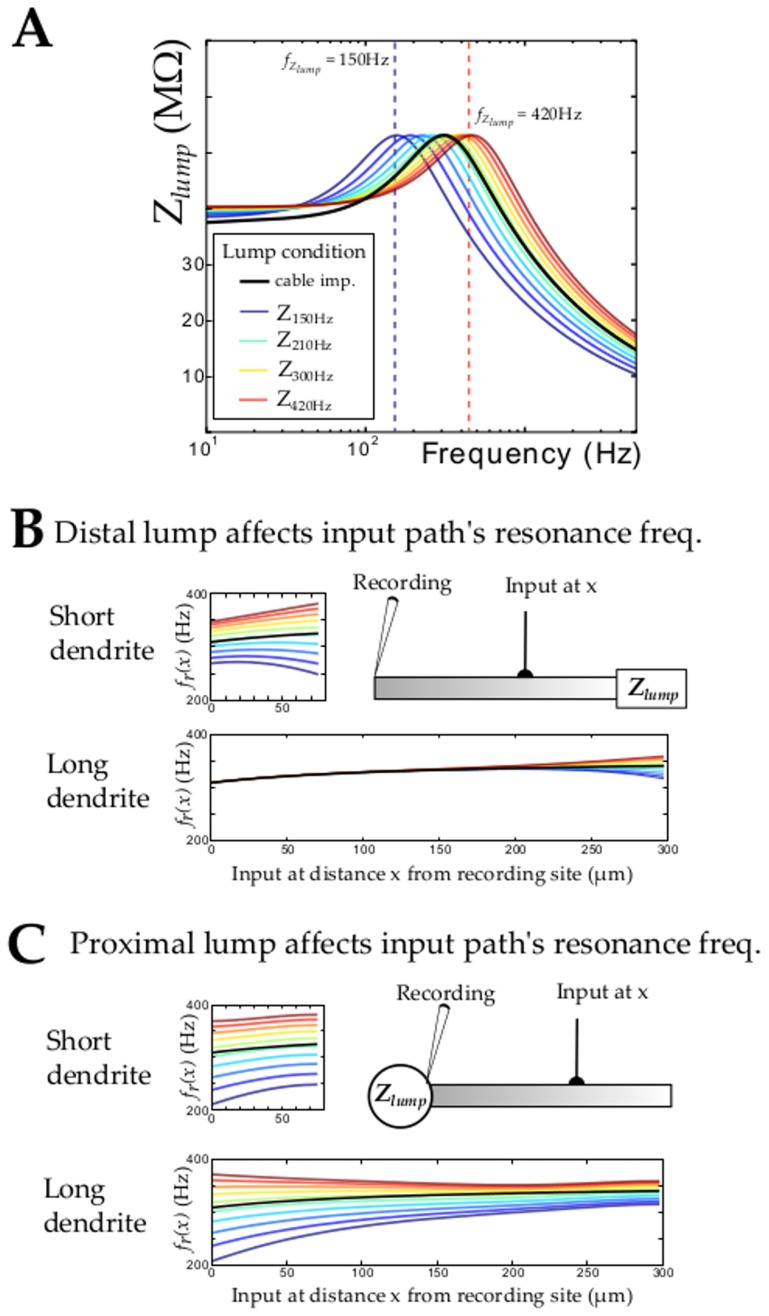
Effect of boundary conditions on the spatial profile of resonance frequencies observed in a dendritic segment. A. To explore the effect of boundary conditions on a dendritic segment, different resonant lumped boundary conditions, 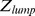, are used. The color-code represents resonance frequency of the “lumped” boundary condition with blue to red corresponding to resonance frequencies ranging lower to higher than the cable characteristics –black line. B. A resonant boundary condition at the tip of a cable mimics sudden changes in membrane parameters and can represents as a first approximation either a change in channel density between segment of a in non-uniform cable or local geometric changes (branching or tapering). The influence of the resonant boundary condition is obtained analytically in the case of this simple abstract morphology. The spatial profile of resonance is shown for the different conditions presented in A. The spatial profile of resonance is influenced over its entire length in the case of short segments (75 µm -upper panel) while long segment (300 µm – lower panel) are affected mostly on their distal tip when compared to the refence case of a semi-infinite cable. C. Similarly, the spatial profile of resonance is drastically changed when a resonant boundary condition is located at the soma. The effect is large and observed over the entire resonant segment, even when the segment is long. This has important implication for e.g., stellate cells for which each dendritic branch “sees” at its proximal ending a resonant “lumped” boundary condition constituted of the soma and all other branches.

A dendrite, however, is structurally far more elaborate than the simplified morphology and uniform membrane of the cable presented so far. Dendritic membranes, for example, often exhibit non-uniform distributions of ionic channels, as well as branching and tapering geometries. To understand such different cases, one can assume as a first approximation that a dendrite is constituted of small uniform cable segments (piecewise constant approximation). The boundary conditions at each end of the uniform segment affect the spatial profile of resonance frequency of the transfer impedance. Therefore, we consider the effects of boundary conditions using a linearized cable model (with parameters similar to Figure 1D,E,F). Figure 2B and C illustrates the spatial profile of resonant frequencies under two geometric configurations: the branching of daughter dendrites at the apical end (Figure 2B) and the attachment of a soma at the basal end (Figure 2C). In both cases, the boundary conditon at the tip of the segment is given by a “lumped” impedance (e.g. representing the impedance of the daugther dendrites lumped together). Moreover, this “lumped” impedance can be set to have different resonance frequencies by varying 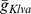, 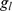, 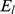. In Figure 2A the “lumped” impedances are presented color coded by their resonant frequency from blue (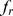 = 150 Hz) to red (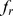 = 420 Hz). The spatial profile produced by each resonant “lumped” impedance is compared to a control condition where the boundary impedance is that of an uniform semi-infinite cable (shown in black in Figure 2A). Compared to the uniform semi-infinite boundary condition, the impedance at the recording location can shift considerably depending on the specific boundary condition and segment dimensions. For example, changes in the resonance frequency of the transfer impedance can be observed throughout the entire length of the segment in the case of a short segment (75 µm) while in the case of a long segment (300 µm) these changes are mainly located close to the modified tip. Interestingly, while boundary conditions modify strongly the profile of resonance frequency, the spatial profile of sharpness is not much affected (see *Supplementary Figure S2*).

**Figure 3:**
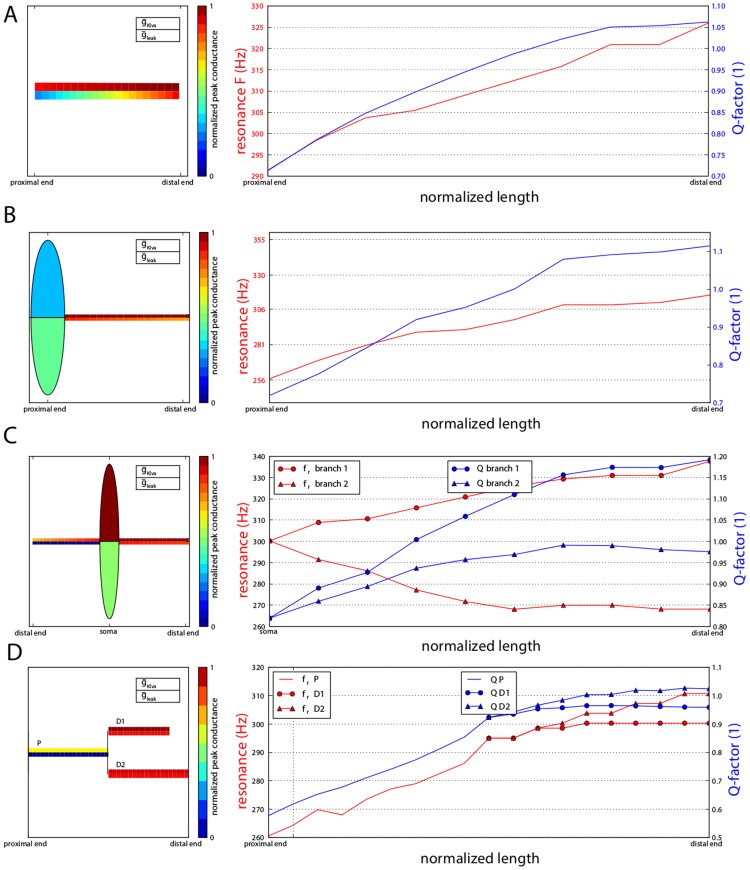
Optimized membrane parameters to achieve the largest range of resonant frequencies with high sharpness (Q-factor). A. Left panel: a sketch of the model cable with non-uniform density of 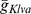 and 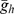 color-coded and normalized to the allowed range (see Methods). Right panel: Optimized range of resonance frequencies (red) and sharpness (blue) along the cable. A gradient of 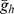 against a constant high density of 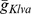 produces the largest frequency range. B. Left panel: The Ball-and-stick model and its optimized conductance density profile. The optimized cable diameter and soma radius are also drawn to scale in the sketch. A similar type of gradient can be observed as in Panel A. Right panel,:Spatial profile of the resonance frequency (red) and sharpness of tuning (blue). **C.** ***Left panel***, bipolar model and non-uniform density of 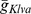 and . Right panel: Spatial profile of resonance frequency (in red) and sharpness of tuning (in blue) with markers indicating the distinct left and right branches. D. Left panel: The “Y-branch” model and its optimized non-uniform density of 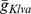 and 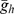. Right panel: Spatial profile of resonance frequency and sharpness of tuning with markers indicating parent (P) and daughter one (D1) and two (D2) in D.

We then investigated the extent to which a spatially nonuniform conductance distribution contributes to the range of resonance frequencies expressed by a neuron. Simulations exploring the distribution of two conductances (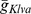 and 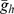) were performed in four types of abstract morphologies: a cable, soma-and-dendrite, bipolar and y-dendrite model (Figure 3). The left panels of Figure 3 A–D provide a schematic of the optimized conductance distribution along the dendrite. Right panels provide the spatial profile of the resonance frequency (red) and sharpness (blue). Optimizing the membrane properties to obtain a large range of resonant frequencies combined with moderate sharpness resulted in specific effects of the non-uniform distribution in each morphology.

For the cable, a gradient of 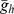 conductances with a constant but high 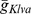 produced the largest range of resonance frequencies as shown in Figure 3A. The spatial gradient of 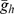 along the cable produces an increasing reversal potential toward its distal tip as well as an increasing total leak (from 0.32 mS/cm^2^ to 1 mS/cm^2^). Both effects tend to raise the input resonance frequency (Supplementary figure S1 A1). Moreover, because of the gradient of 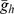, each segment of this non-uniform cable will be connected at its proximal tip to a segment of lower characteristic frequency and at its distal tip, a segment of higher input resonance frequency. This configuration is similar to the configuration of a linear resonant cable producing the largest frequency range along its length (Figure 2 B, C) and the spatial profile of resonance frequency ranges from 292 to 325 Hz. Finally, the density of 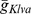 is constant and high (15 mS/cm^2^) and ensures a sharp tuning of input resonance (Supplementary Figure S1, A2). Therefore, the optimization results extend the analytical insights obtained by linearization of the ionic channel dynamics.

A similar gradient is observed in the case of a soma-and-dendrite morphology as depicted in Figure 3B. The density of 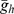 is decreasing from 1 mS/cm^2^ to 0.83 mS/cm. The range of transfer resonant frequencies observed is both caused by the conductance-density gradient the discontinuous boundary condition introduced by the soma (as analyzed in Figure 2). Overall, the increased complexity of the ball-and-stick morphology increased both the range of frequencies expressed (256 to 315 Hz) and the overall sharpness of tuning (<Q> = 0.92) compared to the case of the finite cable shown in figure 3A.The density of 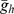 is decreasing from 1 mS/cm^2^ to 0.83 mS/cm^2^. The range of transfer resonant frequencies observed is both caused by the conductance-density gradient the discontinuous boundary condition introduced by the soma (as analyzed in Figure 2). Overall, the increased complexity of the ball-and-stick morphology increased both the range of frequencies expressed (256 to 315 Hz) and the overall sharpness of tuning (<Q> = 0.92) compared to the case of the finite cable shown in Figure 3A.

The optimized conductance profile for the bipolar neuron morphology lead to an even larger range of resonant frequency and Q-factors (Figure 3C). In the bipolar case, the range of transfer resonance frequencies differs in both dendrites mosty due to the different distributions of the leak conductance. In one branch, a low density of both 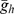 and 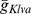 caused relatively low resonance frequencies of the transfer impedance along the branch while a high density in both conductances caused relatively high resonance frequencies in the other branch. As a result, the range of resonance exhibited in the whole neuron was large (between 268 and 338 Hz) and maintained good sharpness (<Q> = 0.99). Thus, thismorphological construct exploited both non-uniform densities and changes in boundary conditions between the soma and each of its two branches.

Similarly, the optimized Y-branch produced a large range of resonance frequencies from its low resonance frequency in the parent branch to the high resonance frequency in the daugther branches (Figure 3D). Thus, dendritic constructs such as branching, tapering and non-uniform channel distributions enrich the spatial distribution of resonant frequencies caused by space alone.

### Neurons as complex spatio-temporal input classifiers

**Figure 4:**
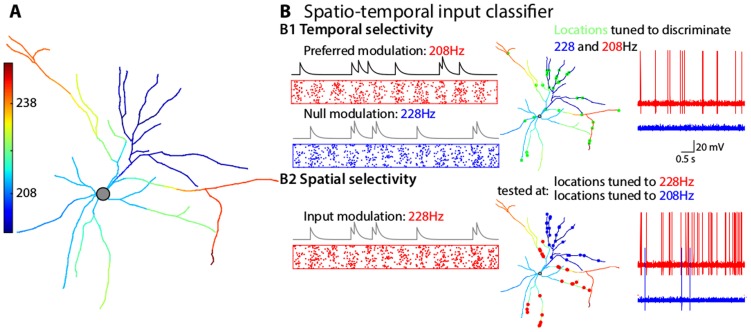
Spatio-temporal input classicifcation in neurons due to to spatial profiles of resonance in the transfer impedance in dendrites. A. Resonance frequencies of the transfer impedance. Each dendritic location is color-coded from blue (207 Hz) to red (247 Hz) and represents the resonance frequencies of the transfer impedance toward the soma (recording location). This demonstrates how non-uniform membrane parameters and a complex multi-polar cell morphology give rise to a large range of spatially segregated temporal filters. B. The spatio-temporal filtering ability of a stellate cell with distributed resonance properties. Two classification tasks are presented: one based on the temporal modulation of the input rate (B1) and one based on the spatial distribution of synapses (B2). In each case, the neuron receives 25 independent non-homogenous Poisson processes inputs. B1. Temporal selectivity: location of the (green) inputs are optimized so that the output spike rate best discriminates between two input signals; a target input signal modulated at 228 Hz and minimized for a null-signal modulated at 208 Hz. A schematic raster plot of the different input signal is shown (red: target signal, blue: null-signal). The target signal triggers many spikes (red trace) while the null-signal triggers none. B2. Spatial selectivity: synapses are inserted at dendritic locations matching a resonance frequency in the transfer impedance of 228 Hz (±4 Hz, red dots) or 208 Hz (±4 Hz, blue dots). When a signal modulated at 228 Hz is presented to the red group of synapses, the neuron responds with many spikes (red trace). When at the same red synapses a singal modulated at 208 Hz is presented, the neuron fails to respond and generates only a few spikes (blue trace).

For a more realistic experimentally reconstructed morphology (downloaded from NeuroMorpho.org, see Methods), the non-uniform distribution of conductances, the complex branching and tapering of dendrites can lead to an even richer spatial distribution of resonance frequency as shown in Figure 4A. We optimized the density of 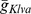 and 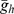 for each branch of this model. Each branch was allowed to have a linear gradient of these two channels and the optimization criteria was to find the model with largest range of resonance frequencies (in the complete neuron) while maintaing a reasonable sharpness (<Q>>0.8, see METHODS). Figure 4A illustrates the model neuron resulting from that first stage of optimization. At each location 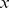 on the dendritic tree, the resonant frequency of 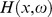 is color-code ranging from 207 Hz (blue) to 247 Hz (red). In this model based on a real morphology, the combination of dendritic geometry and non-uniform ion-channel distribution endow any morphologically realistic model neuron with a rich spatial profiles of resonance. Such spatially distributed and sharply tuned resonance frequencies can effectively act as spatiotemporal filters for a neuron's inputs, which leads us to consider in more detail the functional significance of these resonances. With distinct dendritic locations expressing a preference for certain frequencies, one can envision the dendrite as powerful spatio-temporal filter of synaptic inputs: viewed from the vantage point of the soma, each point on the dendritic tree has a *preferred* input modulation rate that it amplifies while attenuating all others input rates. This is demonstrated by the simulations in Figure 4B where the temporal and the spatial selectivity are illustrated separately (see *Methods*).

*Temporal selectivity* can be demonstrated when one set of synapses (at fixed locations) can cause a differential/preferential response at the soma of the neuron when stimulated with different temporal activation patterns, as illustrated in the scenario of Figure 4B1. Here, the spatial distribution of the green synapses was chosen on the dendritic tree of Figure 4A so as the combined transfer function optimally responds to a 208 Hz modulated spike train while ignoring a 228 Hz input. This simulation demonstrates the dendritic temporal filtering abilities achieved with a combined spatial profile of transfer resonances. Note that in arriving at this result, we did not need to optimize the synapse properties, which are assumed to simply enhance signal transduction to ensure that the frequencies arising on the post-synaptic membrane are near the resonance frequencies shown in panel Figure 4A.

*Spatial selectivity* is illustrated by two sets of synapses at distinct dendritic locations responding differentially to the same signal as shown in Figure 4B2. The red synapses are located at dendritic locations corresponding to a resonance frequency of 228±4 Hz and the blue synapses at 208±4 Hz. When both groups were stimulated separately by Poisson processes modulated at 228 Hz (see Methods), the input at the blue synapses generated only a few spikes at the soma (blue trace). By contrast, the same input signal at the red synapses, elicited many more spikes (red trace). The same signal therefore induced different somatic responses when conveyed to the neuron through distinct sets of synapses with different resonance properties to the soma.

To conclude, a neuron can perform elaborate spatiotemporal filtering of its inputs utilizing the distribution of its dendritic resonances, a capability that is substantially more elaborate than is widely assumed possible of a neuron expressing only one prefered resonant frequency [12], [13], [20].

## Discussion

In summary, building upon the work of Koch and colleagues [19], [21], we have shown that a model of a simple neuronal membrane with typical biophysical properties and ionic channels can readily exhibit a resonant transfer impedance. When viewed from a distance down the cable, the resonance can take a wider range of frequencies and bandwidths. This range expands greatly when considering nonuniform cable models with complex boundary conditions and changing ionic channel densities and types. Finally, the full power and versatility of this dendritic resonance idea comes into focus in a more realistic multi-compartmental model which allowed us to demonstrate its potential functional significance as it enables a neuron to serve as a spatiotemporal filter.

Given the ubiquity and diversity of dendritic resonances, why has their functional significance been thus far neglected? The answer probably lies in the commonly-held view that resonance mainly plays a role in synchrony (and participation therein) at lower frequencies (e.g., α,β, and θ-bands at <10 Hz). At those frequencies it is hard to distinguish experimental variability from a real range of resonance frequencies (a range of 50% around 4 Hz is 2–6 Hz). At the much higher frequencies considered here (and in only one previous report [14]), a 50% range translates to 225–375 Hz. Resonances in those ranges correspond to high gamma. Interestingly, in the lower auditory system, where neurons are known to express fast-activated potassium channels, these higher modulation frequencies can be transmitted by neuron to encode modulation of the sound energy. Temporal modulations at these frequencies convey periodicity cues critical in the perception of pitch [22]. Also, in more central neurons these rates can readily occur in the high-conductance state during which neurons are constantly bombarded with seemingly irregular firing rates [23]. As long as there is a temporal modulation (envelope) rate, dendritic transfer resonance can still filter relevant signals.

It should be pointed that neurons with a rich variety of dendritic transfer resonance may rather be the rule than the exception. Indeed, as we have highlighted here both nonuniform channel conductance and boundary conditions enhance the usual range of transfer resonance expressed by a cable. There have been many studies demonstrating that channels are non-uniformly distributed on the dendrite [24]–[25]. Given that a diverse range of resonances is ubiquitous and inevitable in dendrites, we can speculate on further implications of our findings. A first important observation is the difference between resonant frequencies of the input versus transfer impedance: the input impedance dominates locally while the tranfer impedance is global insofar it spans the complete dendritic membrane along which an input signal travels to the soma. Plasticity can, in principle, differentially exploit local and global effects. At the local level, a signal that temporally matches the resonant frequency in the input impedance may trigger a large local voltage-depolarization giving rise to a calcium transient that, in turn, triggers plasticity mechanisms [26]. At the global level, a different (but not mutually exclusive) hypothesis is based on pre and post-synaptic spike times [27]. In this scenario, the combined synaptic input to a neuron triggers a post-synaptic spike, which then back-propagates into the dendritic tree and activates plasticity mechanisms. Since the strength of somatic depolarizaion depends on the global resonant frequency of the transfer impedance, the most likely inputs to induce spiking (and hence plasticity) are those with modulation rates that match this global resonance.

A slight variation on the latter hypotheses is the case in which a “teacher” signal impinges onto the soma and triggers spikes. In that situation, the neuron can associate the modulation of the “teacher” signal to a specific the set of synapses that have an equal transfer resonance to the soma. Indeed, such a neuron would be responsive only when the preferred modulation rate at the synapses matches that of the teacher signal. Inputs from synapses with transfer resonance modulated at any other rate would not be carried out to the soma and would not interact constructively with the “teacher” signal. This situation is particularly interesting in the auditory system where low frequency cell could provide “teacher” signals to modulation detector neurons with dendritic branches spread across tonotopy (such as octopus cells [28]–[30] or inferior colliculus stellate cells [31]). Since the output modulation rate of low frequency cells is determined by their location, while that of high frequency cell is not, cross-frequency modulation detectors could arise by such a learning of specific input location. This idea provides a neural basis to solve the central problem of linking the rate modulation of low and high frequency places in auditory pitch perception [32].

Thus, resonant frequencies in dendrites not only enable the neurons to perform elaborate spatio-temporal filtering, it can also have pivotal consequences for plasticity, and different plasticity mechanism could be activated by local or global post-synaptic potentials dependent on the temporal signature of the pre-synaptic signal.

## Methods

Neurons are modeled at two different levels in this study: a membrane level (i.e. point neuron) and a compartmental level. Both levels relied on a current-balance equation which describes the ionic flow across the membrane. In addition to the passive flow of current, we focus on one particular restorative voltage-dependent current 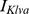 produced by fast activated, slowly inactivating potassium channel. The membrane dynamics are described by:
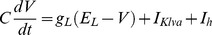
where 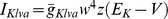 with 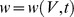 and 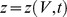 represents the proportion of activated and inactivated ionic channels. Their dynamics are given in the standard form introduced by Hodgkin-Huxley 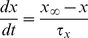, where 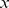 stands for either 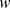 or 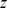. The voltage dependent time constants 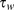, 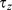 and the activation 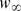 and inactivation 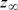 of the potassium channel are taken from Mathews et al. [33]: 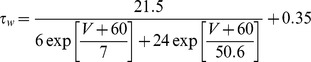, 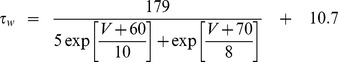, 
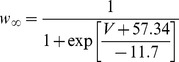
, and 
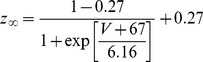
. The time constants parameters and 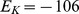 mV are kept constant throughout the study. Because of its much slower time scale, the current 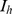 is modeled as a static leak (i.e. 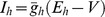) throughout the paper with 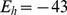 mV.

### Linear analysis of the resonance

The resonance introduced by 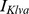 can be described in the Fourier domain [16], [19], [34] after linearizing the current balanced equation around the resting membrane potential 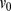. A small variation in the potassium current 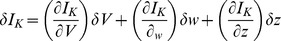 is composed of three terms: an ohmic part (i.e. the steady-state potassium conductance 
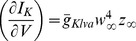
) and two other terms describing the increase and decrease in subsequent changes in activation and inactivation of the channels. The membrane impedance is given by 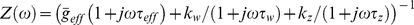, where 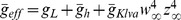 is the effective conductance of the membrane composed of the leak and the steady-state potassium conductance and 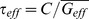 is the effective membrane time constant. The conductance 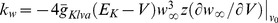 represents the extra conductance associated with opening additional activation gates following a variation of voltage around rest. Correspondingly, 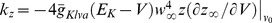 represents the decrease in conductance associated with the closing of some inactivation gates. The frequency dependence of 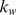 and 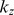 allows a further simplification. Since 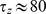 ms [33] while 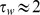 ms, any voltage changes at frequencies above 12.5 Hz have little effect on the inactivation and thus we can neglect effect of the inactivation. Therefore, we use the following expression for the membrane impedance in Figure 1A: 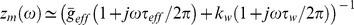.

### Cable model of resonant dendrite

For the spatially extended models (Figure 1D,E and 2), the current-balanced equation for each compartment is similar to that of the membrane with the addition of terms describing the current between compartments which is proportional to the axial resistance 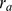. The space constant 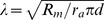 for a dendrite describes the distance between an injection and recording site for which the DC component has decayed of a factor 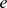. More generally, the membrane impedance 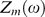 determines the frequency dependent space constant 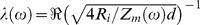, of the dendrite (where 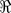 denotes the real part of a complex number). The transfer impedance 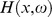 between any two points separated by a distance 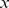 can be computed by solving the generalized cable equation given in the Fourier domain by 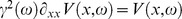 with its appropriate boundary conditions, where 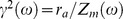. For the semi-infinite cable described in Figure 1, its magnitude reads 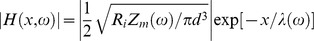 and this was used to compute the spatial profile of the resonant frequency 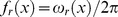 and spatial profile of Q-factor, denoted 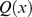 (see below). The space constant of the semi-inifinite cable is thus related to input impedance by 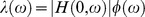 where 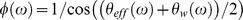 with 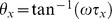. This relationship demonstrates why an inherent mismatch exists between the resonance frequency of the space constant is different than that of the the input impedance. When more specific boundary conditions are used (Figure 2), the transfer impedance 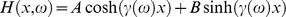 does not easily relate to the concept of space constant. Different approaches [21], [35], [36] can be used to compute 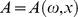 and 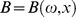 from the boundary conditions. We have used the expression of rule I and III of Koch and Poggio [21].

### Compartmental model of resonant dendritic tree

Numerical simulations to determine the influence of complex dendritic morphologies on resonance were performed using the NEURON+Python [37], [38] software. In order to explore the wide range of parameters that leads to significant spatio-temporal input filtering, we performed evolutionary optimizations [39], [40] of abstract (cable, bipolar, multipolar, “Y” dendrites) model neurons (Figure 3) as well as morphological detailed model neurons (see Figure 4). Optimization by evolutionary algorithms involved two critical steps: parametrization of the model neurons so they can be systematically optimized and, the quantitative assessment of the models to guide the optimization.

The parameters used for the optimization are summarized in Table S1. These parameters are based on neurons from the early auditory pathway [31], [41]–[43]. Note that in each of these models the segment diameters as well as the conductance densities may follow a linear gradient between an initial and ending value. The diameter is additionally constrained not to increase. The length of the dendritic branches in the abstract models is adjusted so that the total length of the path between soma and termination point is 200 micron.

The quantitative assessement of the models we are established by two means. First, the spatial profile of resonance frequency 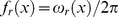 allows us to compare quantitatively the range of frequencies obtained on a fixed morphology. For the linear cable, this is obtained by numerically computing 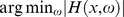. For the compartmental model with nonlinear channel dynamics, an “impedance amplitude profile”- current (ZAP-current [44]) is injected at a specific location in the dendritic segment and the frequency at which the membrane potential is maximal (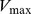) is taken as the resonant frequency (i.e. 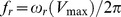). The second assemement is based on the sharpness of tuning, also called the Q-factor. Rather than defining the Q-factor by 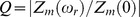, as done in various study [12], [19], [45], we use a definition focusing on the bandpass properties offered by dendritic resonance, that is: how quickly the resonant response drops around the resonant frequency 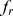. The Q-factor is thus defined by 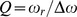 where 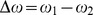 denotes the bandwidth of the resonance and 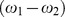 are such that 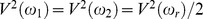. The spatial profile of the Q-factor, 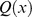 is determined by computing Q at each point along the dendrite.

We can then decide to optimize for range of resonance frequencies obtained, the overall Q factor or both Simultaneously (as in Figure 3).

### Spatio-temporal input filtering on realistic spiking model of neuron

To demonstrate the spatio-temporal filtering in a spiking model with a realistic morphology, a neuron model with an archetypical multipolar morphology [46] (“P2-DEV139” originally published in [44] available at the NeuroMorpho.org archive [47]) is simulated and optimized. We optimize this model neuron in two steps. First, the membrane properties (Table S1) are modified iteratively to obtain a large range of resonance frequencies (resulting in a 207 to 247 Hz range – see Figure 3A) and with reasonable sharpness in the dendrites (0.79<Q<0.89). Second, while using these optimal membrane parameters, we optimize synaptic parameters and input parameters for two tasks: temporal or spatial filtering. Both tasks exemplify the single property of the optimized neuron, namely to perform spatio-temporal input classification. For both tasks, the synaptic input parameters optimization is performed as follows. Inputs spike trains onto 25 synapses are obtained from independent non-homogeneous Poisson processes (NHPP) with sinusoidal firing rate 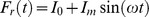 where 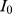 and 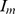 are both optimization parameters. A DC current is added to the soma segment representing the global background activity. To demonstrate the temporal selectivity, we fix the modulation frequency 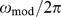 to a target frequency (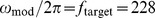 Hz) or a null frequency (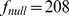 Hz). The synapses' location and strength is optimized for a discrimination task: output spike rate is maximized for 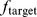 and minimized for 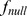, that is, the location and strength is kept identical for the two different inputs (figure 3B1, green dots). Because the synaptic locations are the same in both cases, the neuron can only use temporal information of the input to filter the target from the null signal. To demonstrate the spatial selectivity, we fix the input frequency at 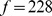 Hz and optimize synapses' location and strength for two different sets of synapses: the “target set” which should maximize the output firing rate and the “null set” which is optimized for a different frequency. Because the input signal is identical in both cases, the neuron can only use the location of the synapse to filter one signal but not the other (Figure 3, B2)

## Supporting Information

Figure S1Membrane conductance parameters affect both input and transfer resonance. **A1.** Resonance frequency of the input impedance depends on both the potassium and leak conductances (respectively, 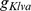 and 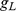). At potassium conductance 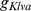 larger than 10 msiem./cm^2^ the resonance frequency is closely related to the effective cutoff frequency 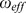. **A2.** The quality factor of the input impedance is in part determined by 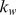 although a region of high sharpness of tunning is found around 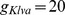 msiem./cm^2^ and 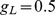 msiem./cm^2^. **B.** The transfer impedance has a different resonance frequency depending on the location of the input (see Figure 1C, D). The mismatch (**B1**) between 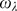 (resonance frequency of the cable space constant 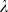) and 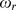 (resonance frequency of the membrane impedance) explains the range of resonance frequency seen along a semi-infinte cable(**B2**).(TIF)

Figure S2Influence of dendritic structure on the spatial profile of the Q-factor of the transfer impedance. **A.** Different resonant lumped boundary conditions, 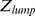, are color-coded with blue representing boundaries with lower resonance frequencies and red higher. Black describes the case of the uniform semi-infinite cable. **B.** A resonant lump at the tip of a cable mimics sudden changes in membrane parameters. The influence of the lump is obtained analytically in the case of this simple abstract morphology. The spatial profile of Q-factor is shown for the different 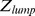 presented in A. A short and a long segment are displayed to show that the sharpness of tuning is not affected much compared to the refence case of a semi-infinite cable. This observation is also valid in the case of a resonant lump at the soma (**C**) for which important changes in resonance frequency can be observed (Figure 2C).(TIF)

Table S1Parameters of the conductance based model subject to optimization (Figure 3–4). Allowed values for the parameters must be inside the given ranges. Default values are inspired by auditory nucleus neurons that contain the fast 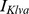. For the full morphology the membrane resistance was increased to resemble that of a neocortical cell.(DOCX)
