# Supplementary figures and images for: Spatially Distributed Dendritic Resonance Selectively Filters Synaptic Input
Source: PLoS Comput Biol. 2014 Aug 21;10(8):e1003775. doi: 10.1371/journal.pcbi.1003775 (PMC4140644; doi:10.1371/journal.pcbi.1003775)

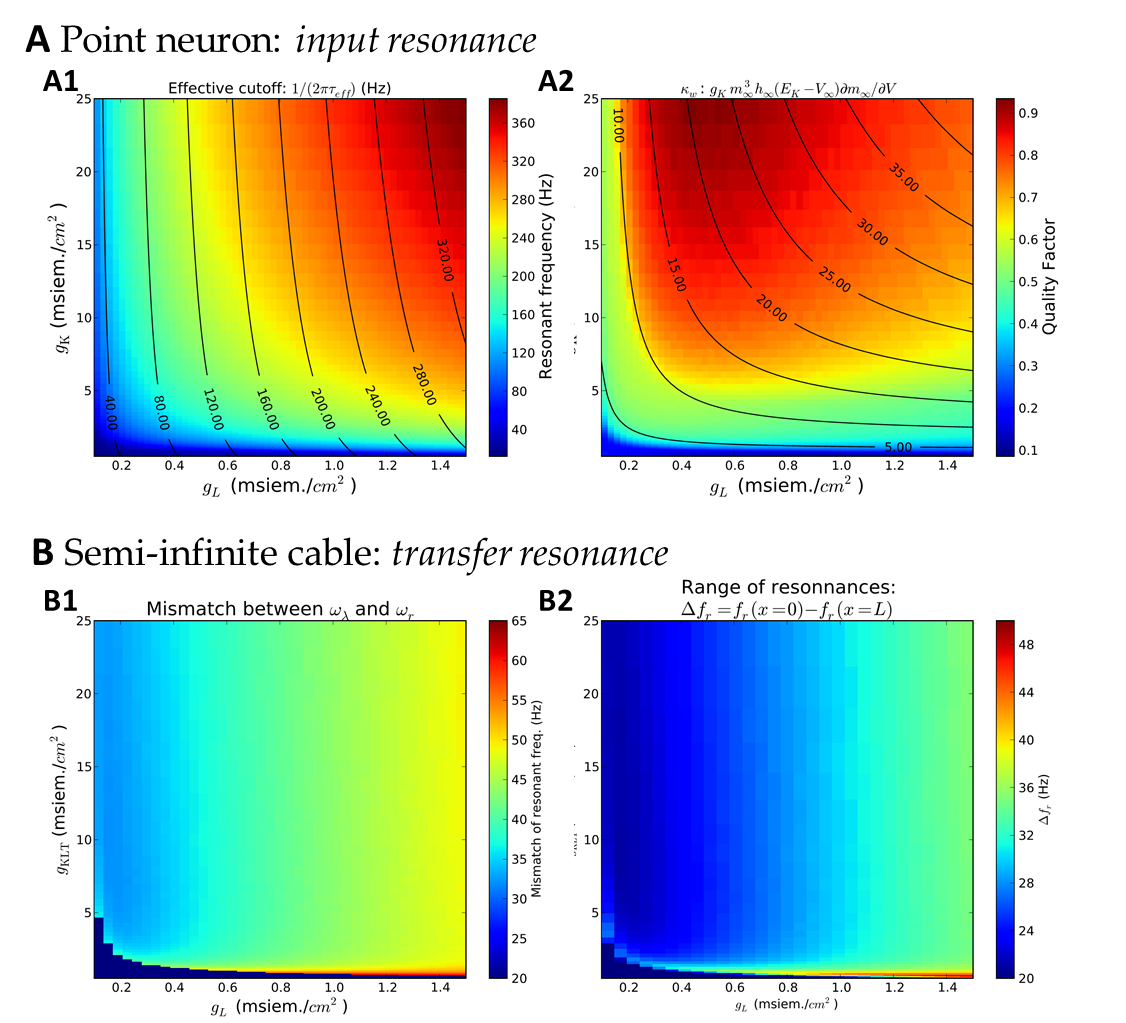

Supplement: Figure S1 — Membrane conductance parameters affect both input and transfer resonance. A1. Resonance frequency of the input impedance depends on both the potassium and leak conductances (respectively, and ). At potassium conductance larger than 10 msiem./cm2 the resonance frequency is closely related to the effective cutoff frequency . A2. The quality factor of the input impedance is in part determined by although a region of high sharpness of tunning is found around msiem./cm2 and msiem./cm2. B. The transfer impedance has a different resonance frequency depending on the location of the input (see Figure 1C, D). The mismatch (B1) between (resonance frequency of the cable space constant ) and (resonance frequency of the membrane impedance) explains the range of resonance frequency seen along a semi-infinte cable(B2). (TIF) [file pcbi.1003775.s001.tif]

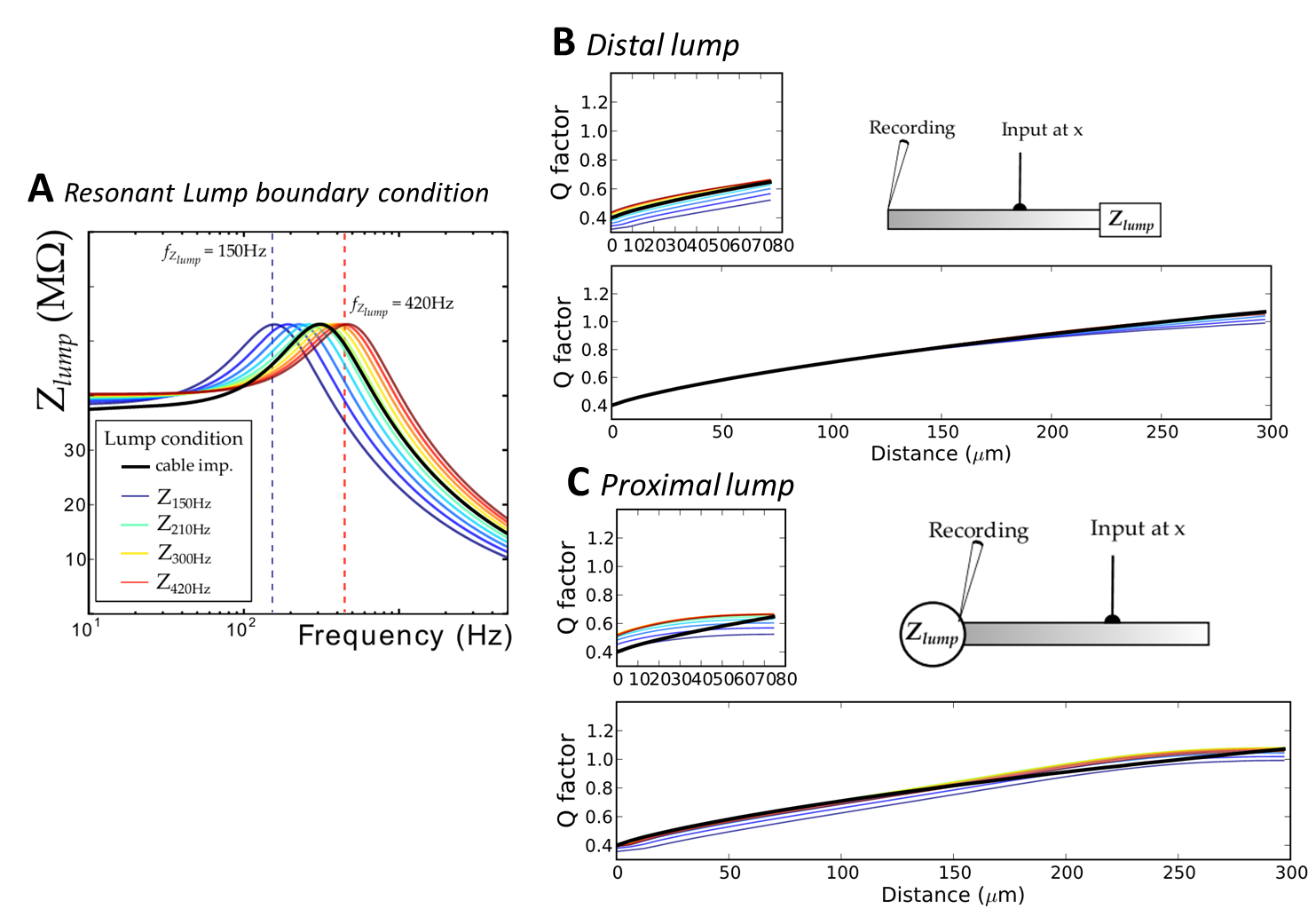

Supplement: Figure S2 — Influence of dendritic structure on the spatial profile of the Q-factor of the transfer impedance. A. Different resonant lumped boundary conditions, , are color-coded with blue representing boundaries with lower resonance frequencies and red higher. Black describes the case of the uniform semi-infinite cable. B. A resonant lump at the tip of a cable mimics sudden changes in membrane parameters. The influence of the lump is obtained analytically in the case of this simple abstract morphology. The spatial profile of Q-factor is shown for the different presented in A. A short and a long segment are displayed to show that the sharpness of tuning is not affected much compared to the refence case of a semi-infinite cable. This observation is also valid in the case of a resonant lump at the soma (C) for which important changes in resonance frequency can be observed (Figure 2C). (TIF) [file pcbi.1003775.s002.tif]
