# Supplementary material for: Spatially Distributed Dendritic Resonance Selectively Filters Synaptic Input
Source: PLoS Comput Biol. 2014 Aug 21;10(8):e1003775. doi: 10.1371/journal.pcbi.1003775 (PMC4140644; doi:10.1371/journal.pcbi.1003775)
Supplement: Table S1 — Parameters of the conductance based model subject to optimization (Figure 3–4). Allowed values for the parameters must be inside the given ranges. Default values are inspired by auditory nucleus neurons that contain the fast . For the full morphology the membrane resistance was increased to resemble that of a neocortical cell. (DOCX) [file pcbi.1003775.s003.docx]

Supplementary Table S1

| Parameter | “Default” cable | Cable model | Bi-polar model | “Y-dendrite” | Full morphology |
| --- | --- | --- | --- | --- | --- |
| Rm ($\Omega/cm^{2}$) | $[3000,10000]$ | $[3000,10000]$ | $[3000,10000]$ | $[3000,10000]$ | $[20000,30000]$ |
| Cm ($pF$) | $[0.8,1.1]$ | $[0.8,1.1]$ | $[0.8,1.1]$ | $[0.8,1.1]$ | $[0.8,1.1]$ |
| Ra ($\Omega/cm^{2}$) | $[100,250]$ | $[100,250]$ | $[100,250]$ | $[100,250]$ | $[100,250]$ |
| $E_{L}$ ($mV$) | $[-70,-50]$ | $[-70,-50]$ | $[-70,-50]$ | $[-70,-50]$ | $[-70,-50]$ |
| soma L (= diam) ($\mu m$) |  |  | [5,25] |  |  |
| soma $g_{k}$ ($mS/cm^{2}$) |  |  | $[1,25]$ |  | $[1,25]$ |
| soma $g_{h}$ ($mS/cm^{2}$) |  |  | $[0.1,1.0]$ |  | $[0.1,1.0]$ |
| segment L ($\mu m$) | $[20,200]$ | $[20,200]$ | $[20,200]$ | $[20,100]$ |  |
| segment D ($\mu m$) | $[1,5]$ | $[1,5]$ | $[1,5]$ | $[1,5]$ |  |
| segment $g_{k}$ ($mS/cm^{2}$) | $[1,25]$ | $[1,25]$ | $[1,25]$ | $[1,25]$ |  |
| segment $g_{h}$ ($mS/cm^{2}$) | $[0.1,1.0]$ | $[0.1,1.0]$ | $[0.1,1.0]$ | $[0.1,1.0]$ |  |
